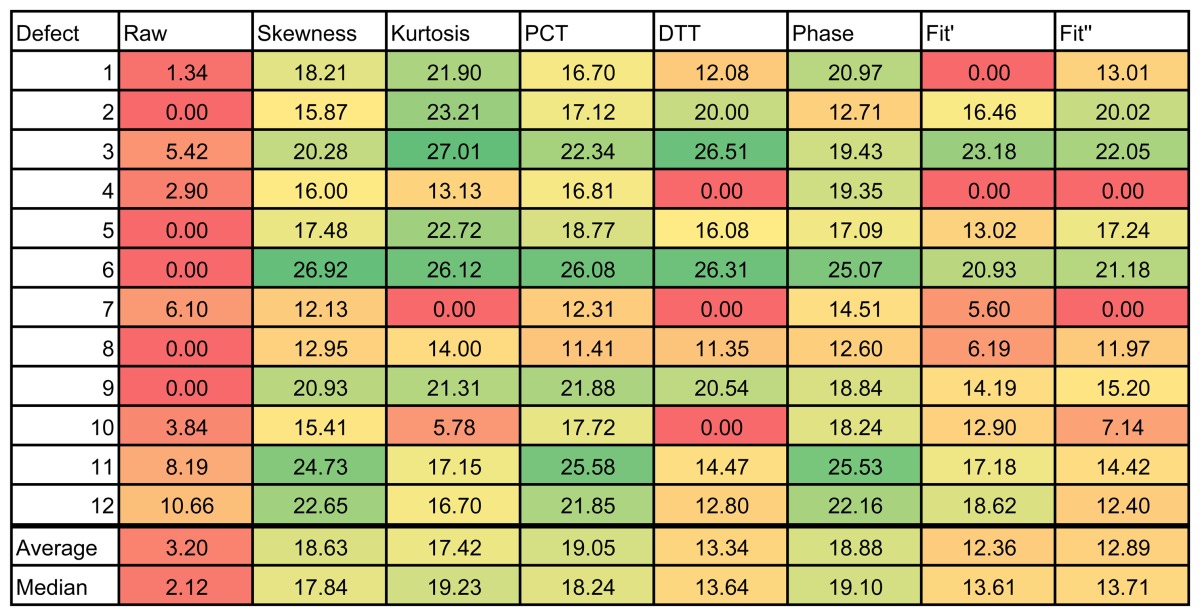# Infrared Thermography for Temperature Measurement and Non-Destructive Testing

**DOI:** 10.3390/s140712305

**Published:** 2014-07-10

**Authors:** Rubèn Usamentiaga, Pablo Venegas, Jon Guerediaga, Laura Vega, Julio Molleda, Francisco G. Bulnes

**Affiliations:** 1 Department of Computer Science and Engineering, University of Oviedo, Campus de Viesques 33204 Gijón, Asturias, Spain; E-Mails: jmolleda@uniovi.es (J.M.); bulnes@uniovi.es (F.G.B.); 2 Aeronautical Technology Centre (CTA), Parque Tecnológico de Álava, Juan de la Cierva 1, 01510 Miñano, Spain; E-Mails: pablo.venegas@ctaero.com (P.V.); jon.guerediaga@ctaero.com (J.G.); laura.vega@ctaero.com (L.V.)

**Keywords:** infrared thermography, active thermography, passive thermography, temperature measurement, non-destructive testing

## Abstract

The intensity of the infrared radiation emitted by objects is mainly a function of their temperature. In infrared thermography, this feature is used for multiple purposes: as a health indicator in medical applications, as a sign of malfunction in mechanical and electrical maintenance or as an indicator of heat loss in buildings. This paper presents a review of infrared thermography especially focused on two applications: temperature measurement and non-destructive testing, two of the main fields where infrared thermography-based sensors are used. A general introduction to infrared thermography and the common procedures for temperature measurement and non-destructive testing are presented. Furthermore, developments in these fields and recent advances are reviewed.

## 1. Introduction

Infrared thermography (IRT) is a science dedicated to the acquisition and processing of thermal information from non-contact measurement devices [1]. It is based on infrared radiation (below red), a form of electromagnetic radiation with longer wavelengths than those of visible light. Any object at a temperature above absolute zero (*i.e.*, *T* > 0 *K*) emits infrared radiation [2]. The human eye cannot see this type of radiation. Thus, infrared measuring devices are required to acquire and process this information [3].

Infrared measuring devices acquire infrared radiation emitted by an object and transform it into an electronic signal [4]. The most basic infrared device is a pyrometer, which produces a single output using a single sensor. Most advanced devices include an array of sensors to produce a detailed infrared image of the scene. The difference between a visible image and an infrared image is that the visible image is a representation of the reflected light on the scene, whereas in the infrared image, the scene is the source and can be observed by an infrared camera without light. Images acquired using infrared cameras are converted into visible images by assigning a color to each infrared energy level. The result is a false-color image called a thermogram [5].

IRT has many advantages over other technologies [6]. In general, the main advantages of IRT are the following:

- IRT is a non-contact technology: the devices used are not in contact with the source of heat, *i.e.*, they are non-contact thermometers. In this way, the temperature of extremely hot objects or dangerous products, such as acids, can be measured safely, keeping the user out of danger.
- IRT provides two-dimensional thermal images, which make a comparison between areas of the target possible.
- IRT is in real time, which enables not only high-speed scanning of stationary targets, but also acquisition from fast-moving targets and from fast-changing thermal patterns.
- IRT has none of the harmful radiation effects of technologies, such as X-ray imaging. Thus, it is suitable for prolonged and repeated use.
- IRT is a non-invasive technique. Thus, it does not intrude upon or affect the target in any way.

IRT provides particular advantages in the medical and veterinary field, as it provides accurate readings without invasive procedures. The fact that IRT is a non-contact technique is very important in this field, because this means that it is a painless procedure. Moreover, as the instrument is non-contact, it does not affect the result of the measurement and can be carried out remotely. Other alternatives can be painful, and when the sensor is in contact with the animal, it can affect the measurement. Therefore, IRT is very effective, not only for measuring the temperature of the animal, but other variables, such as stress [7].

Due to all of these advantages, thermography has been established as an effective tool in many different applications [8]. However, IRT is not without its drawbacks. Fast and affordable hardware has recently become available, but an infrared camera is still an expensive device. Some very affordable models with a high enough spatial resolution for most applications (640 × 512) have recently come onto the market. However, these inexpensive models with high spatial resolution provide lower accuracy, which makes them unusable for some applications. Infrared images can also be difficult to interpret; in general, specific training is required. IRT is also highly dependent on working conditions, such as the surrounding temperature, airflow or humidity. Therefore, IRT must be used in controlled environments.

The intensity of the infrared radiation emitted by objects is mainly a function of its temperature; the higher the temperature, the greater the intensity of the emitted infrared energy. Many different applications can take advantage of this feature [9], from the temperature control of domestic induction cook tops [10], to mobile-robot positioning in intelligent spaces [11], to name but two recent applications. Some of the main fields where infrared thermography is used include medicine [12], veterinary medicine [13], maintenance and process monitoring [14], building inspection [15] and non-destructive testing [16].

Temperature is a very good indicator of health, as changes of just a few degrees on skin (cutaneous or superficial) can be used as an indicator of possible illness [17]. Thus, medical applications use IRT as an alternative diagnostic tool. For example, IRT is used to detect superficial body tumors, such as breast cancer [18]. Tumors generally have an increased blood supply that increases the skin temperature over them [19]. Therefore, IRT can be used as an effective early indicator of breast cancer [20], which results in a much higher chance of survival [21]. In these applications, IRT is a complementary diagnostic tool with high efficiency only in the detection of early warning signals. This early detection is the main advantage of IRT compared with other methods. IRT is used in many other medical applications, such as the diagnosing of diabetic neuropathy or vascular disorders [22], fever screening [23], skin diseases [24], dentistry and dermatology [25] and heart operations [26].

Maintenance is another area where IRT is successfully applied [27]. The electrical field, the mechanical field and insulation are three of the most common areas where IRT is used. IRT is used in electrical and mechanical maintenance to detect early signs of malfunction, so costly breakdowns can be avoided. In the electrical field, abnormal temperature patterns can indicate faulty connections [28], whereas in the mechanical field, they can indicate excessive friction due to improper lubrication or material fatigue [29,30]. In the insulation field, IRT is used to detect hidden losses of heat that can drain performance and increase costs [31]. IRT is also used in other areas of the maintenance and process monitoring field, such as monitoring of plastic deformations [32], evaluation of fatigue damage in materials [33] and weld inspection [34].

Another area where IRT is successfully applied is building inspection. The temperature distribution on the facade of a building provides very useful information to discover many hidden conditions related to the building performance and maintenance [35]. For example, it can be used to detect where and how energy is leaking from a building envelope. Besides the detection of heat loss, IRT is also used to discover other anomalies, such as water infiltration and moisture [36,37]. A wet mass in a wall has a differentiated thermal inertia that can be discovered using IRT. Recent applications of IRT for moisture detection can be found in [38] and [39], which use IRT for sub-surface moisture detection in masonry structures and for moisture mapping in ancient buildings, respectively. Moisture detection using IRT is not limited to buildings. It can also be applied to paper [40], soil [41] or aircraft structures [42]. The presence of water inside aircraft structures may lead to ice formation with a volume variation and consequent mechanical stresses.

Two different approaches are employed in IRT: passive and active [43]. In passive IRT, the radiation coming from the target object is measured without any external heat stimulation. This information can be used for temperature measurement. On the other hand, in active IRT, the specimen is subjected to external thermal stimulation [44,45]. The heat propagation depends on the material's thermal properties, but also on subsurface anomalies, which result in temperature differences on the surface target. In this case, the measured radiation comes from the thermal response of the target to the external excitation.

Passive IRT is used in quality control and process monitoring applications. Temperature plays a crucial role in any industrial process. Thus, temperature measurement and monitoring during and after the industrial process is critical to achieve optimal results, such as steel rolling or sinterization. However, the computation of temperature from infrared images is not only based on measured radiation; it also depends on the internal camera calibration, as well as on the emissivity of the object radiating energy. Thus, a calibration setup is required to obtain accurate measurements.

Active IRT is mostly used in non-destructive testing applications, where an external stimulus is applied to the specimen in order to induce relevant thermal contrasts between regions of interest [46]. It is applied to the inspection of materials for subsurface defect detection and also to detect areas of the specimen with different properties below the surface. Some subsurface anomalies are very subtle. Therefore, the signal levels associated with them can be lost in the thermographic data noise [47]. In these cases, different post-processing methods can be used to improve the signal-to-noise (SNR) content of thermographic data.

This review focuses on IRT for temperature measurement and non-destructive testing, two of the main fields where IRT-based sensors are used. This work is organized as follows. Section 2 presents the principles and essential theoretical background of IRT. Section 3 describes how temperature measurement is carried out using IRT and the measurement setup. Some examples of image processing methods and data analysis are presented and discussed. Section 4 presents a review of non-destructive testing using IRT, how this field has evolved in recent years and advances in the field.

## 2. Principles of Infrared Thermography

Infrared radiation is the energy radiated by the surface of an object whose temperature is above absolute zero [48]. The emitted radiation is a function of the temperature of the material; the higher the temperature, the greater the intensity of the infrared energy emitted.

There are three ways by which the radiant energy striking an object may be dissipated: absorption, transmission and reflection [49]. The fractions of the total radiant energy that are associated with each of these modes of dissipation are referred to as the absorptivity, transmissivity and reflectivity of the body [50]. Three parameters are used to describe these phenomena: the spectral absorptance *α*_λ_, which is the ratio of the spectral radiant power absorbed by the object, the spectral reflectance *ρ*_λ_, which is the ratio of the spectral radiant power reflected by the object, and the spectral transmittance *τ*_λ_, which is the ratio of the spectral radiant power transmitted by the object. These three parameters are wavelength dependent. The sum of these three parameters must be one at any wavelength, as in Equation (1):

(1) $$\alpha_{\lambda }+\rho_{\lambda }+\tau_{\lambda }=1$$

In the case of opaque materials, Equation (1) is simplified as Equation (2), that is, all of the striking energy is either absorbed or reflected. It could also be said that the striking energy that is not absorbed is reflected.

(2) $$\alpha_{\lambda }=1-\rho_{\lambda }$$

Materials in which the transmissivity and the reflectivity are null are called blackbodies. In these materials, all of the striking radiant energy is absorbed (*α*_λ_ =1).

Electromagnetic radiation emitted from a blackbody (*W*_λ_*_b_*) can be calculated using Planck's law, as in Equation (3), where *C*_1_ and *C*_2_ are constants, λ is the wavelength and *T* is the temperature. The result of Plank's law is the power emitted per unit area per unit wavelength, which is a function of λ and *T*.

(3) $$W_{\lambda b}=\frac{C_{1}\lambda^{-5}}{e^{\frac{C_{2}}{\lambda T}}-1}$$

**Figure 1.**
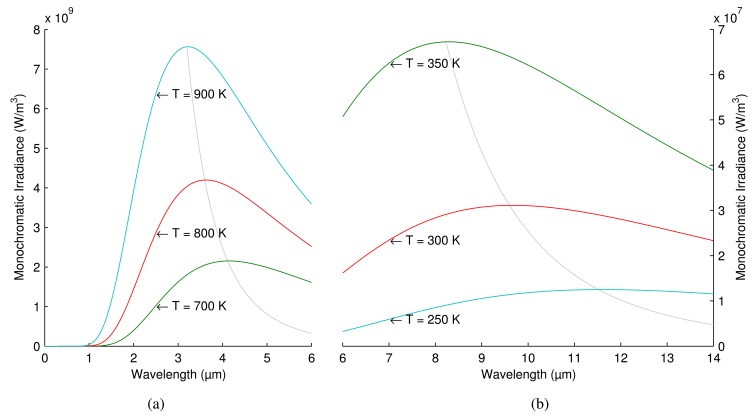
Planck's law: electromagnetic radiation emitted by a blackbody in thermal equilibrium at a definite temperature. (**a**) Objects with a high temperature emit most of the radiation in the middle wave infrared; (**b**) Objects with a low temperature emit most of the radiation in the long wave infrared. The two parts of the graph are scaled differently on the *y*-axis.

Figure 1 shows the distribution of electromagnetic radiation emitted by a blackbody at different temperatures. The curves show how much energy is radiated at each wavelength. As can be seen, the peak of the curve for a hotter object is larger. In addition, there is an inverse relation between the temperature and the wavelength of the peak of the emission.

**Figure 2.**
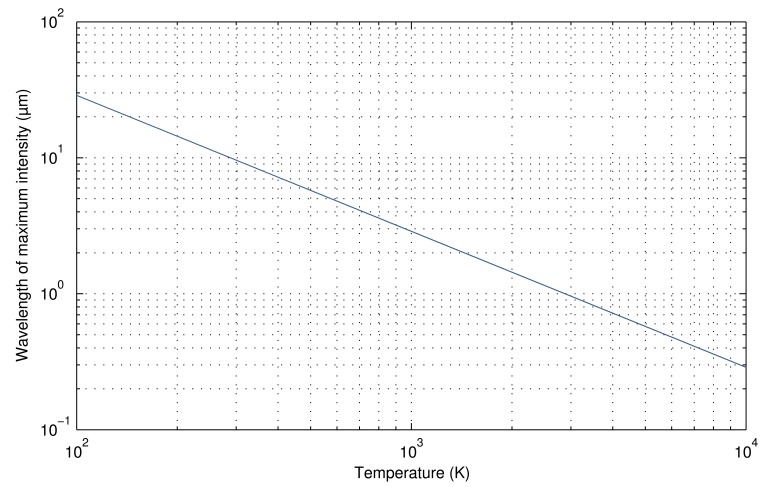
Wien's displacement law: the wavelength of the maximum radiation intensity by a blackbody at a given temperature.

The wavelength at which electromagnetic radiation is emitted depends on the temperature of the object; the higher the temperature, the shorter its wavelength. The distribution is similar, but the wavelength is displaced. The peak wavelength for a specific temperature value can be calculated using Wien's law, as Equation (4). Figure 2 shows a graphical representation of the relation between peak wavelength and temperature using a logarithmic scale. Wien's law is obtained by differentiating the Planck's law Equation (3) with respect to λ and by finding the maximum radiation intensity.

(4) $$\lambda_{\text{peak}}=\frac{0.0029}{T}$$

In order to obtain the total hemispherical radiation intensity of a blackbody, Equation (3) is integrated through all wavelengths (λ from zero to infinity), obtaining Equation (5), where *σ* is a constant. This is called the Stefan–Boltzmann formula.

(5) $$W_{b}=\sigma \cdot T^{4}$$

The emissivity of a body is defined formally for a wavelength λ by Equation (6), as the ratio of the radiant energy emitted by the body to the radiation that would be emitted by a blackbody at the same temperature.

(6) $$\varepsilon_{\lambda }=\frac{W_{\lambda }}{W_{\lambda b}}$$

A real body emits only a fraction of the thermal energy emitted by a blackbody at the same temperature. If the emissivity is constant and independent of the wavelength, the body is a greybody. Thus, it can be expressed as Equation (7):

(7) $$\varepsilon_{\lambda }=\frac{W_{\lambda }}{W_{\lambda b}}=\frac{W}{W_{b}}=\varepsilon$$

The emissivity of real objects is not constant nor independent of the wavelength; thus, they cannot be considered greybodies. However, it is usually assumed that for short wavelength intervals, the emissivity can be considered as a constant. This assumption is used to treat real objects as greybodies. Thus, although the emissivity of real objects is wavelength dependent, and, therefore, they cannot be considered true greybodies, they are treated as such by averaging their emissivity through short intervals, in which the infrared sensor works. This average is also possible because the emissivity is a slow-varying function of wavelength for solid objects. However, this does not apply to other cases, such as gases or liquids.

**Figure 3.**
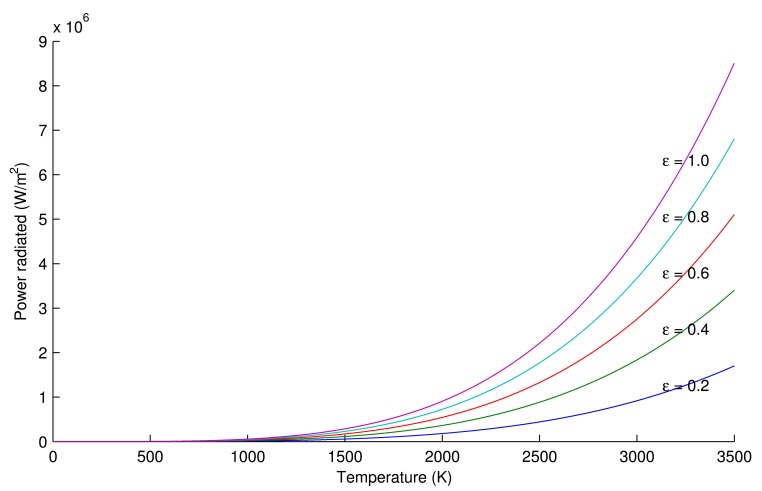
Stefan–Boltzmann law: power radiated by a greybody with different emissivities.

Substituting (7) in (5), (8) is obtained. This equation is the Stefan–Boltzmann formula for greybody radiators. Figure 3 shows a graphical representation of this formula for different emissivities.

(8) $$W=\varepsilon \;\cdot \;\sigma \;\cdot \;T^{4}$$

If all of the radiation energy falling on an object is absorbed (no transmission or reflection), the absorptivity is one. At a steady temperature, all of the energy absorbed must be re-radiated (emitted), so that the emissivity of such a body would be one. Therefore, the absorptivity in a blackbody is equal to emissivity, which is one. In general, according to Kirchhoff's law, the emissivity and absorptivity of any material are equal at any specified temperature and wavelength. This can be expressed as Equation (9):

(9) $$\varepsilon_{\lambda }=\alpha_{\lambda }$$

From Equations (9) and (2), Equation (10) is obtained for opaque materials.

(10) $$\rho_{\lambda }=1-\varepsilon_{\lambda }$$

Greybodies emit only a fraction of the thermal energy emitted by an equivalent blackbody; therefore, emissivity in these bodies is always less than one and reflectivity greater than zero.

### 2.1. Bands

The electromagnetic spectrum is divided into several regions or bands according to the wavelength. Regions are not sharply defined, and they differ in different disciplines. The infrared region is approximately defined from 0.8 μm to 1000 μm, that is, from the end of visible light to microwaves.

Much of the infrared range of the electromagnetic spectrum is not useful in IRT, because it is blocked by the atmosphere. The remaining portions define the usable part of the infrared by IRT:

- Near-infrared (NIR) from 0.8 μm to 1.7 μm.
- Short-wavelength infrared (SWIR) from 1 μm to 2.5 μm.
- Mid-wavelength infrared (MWIR) from 2 μm to 5 μm.
- Long-wavelength infrared (LWIR) from 8 μm to 14 μm.

Of all of these regions, MWIR and LWIR are the most commonly used in IRT. There are two reasons: the band of peak emissions and atmospheric transmittance. The first reason is due to the relation between temperature and wavelength. The most effective measurement for a particular temperature should be carried out for the wavelength at which most intensity is emitted (see Figure 1). Measuring at a different wavelength would require a much more sensitive camera to achieve identical performance. Thus, for most applications, wavelengths longer than SWIR are required. The second reason is related to the atmospheric transmittance. Infrared radiation travels through air, being absorbed by various air particles, mostly by CO_2_ and H_2_O [3]. The degree to which air absorbs infrared radiation depends on the wavelength. In the MWIR and LWIR bands, this absorption is low, allowing more radiation to reach the sensor of the camera.

**Figure 4.**
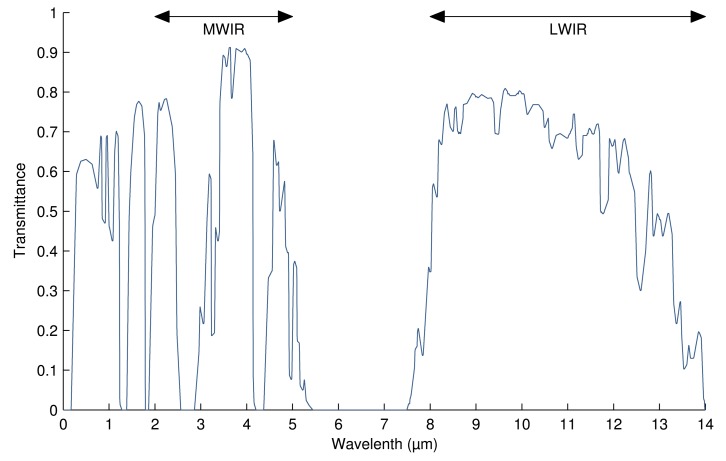
Atmospheric transmittance at one nautical mile, 15.5 °C, 70% relative humidity and at sea level.

Figure 4 shows the atmospheric transmittance for different wavelengths. As can be seen, in the visible part of the spectrum, from 0.4 μm to 0.7 μm, only 60% of the emitted radiation is transmitted. However, between 5 μm and 7.5 μm, almost no radiation is transmitted. The atmosphere absorbs all of this radiation. Therefore, infrared measuring devices use either MWIR or LWIR. MWIR devices are used for high-temperature readings, while LWIR is used for ambient temperatures.

### 2.2. Digitization

Digitization begins with a sensor or detector that transforms infrared radiation into an electronic signal [51]. The detector provides a voltage proportional to the received radiation. The most common can be classified as belonging to two main families: uncooled microbolometric detectors and cooled detectors used for high sensitivity cameras. These detectors are based on different technologies, such as InSb, InGaAs, PtSi, HgCdTe and layered GaAs/AlGaAs for quantum well infrared photon (QWIP) detectors. A review of infrared sensors can be found in [52] and an evaluation in [53]. A detailed historical evolution of these devices can be found in [54].

There are two types of infrared cameras: single sensor and focal plane array (FPA). In order to obtain two-dimensional measurements, single sensor cameras use a rotating mirror. The sensor measures the irradiated energy over the surface of an object reflected on the mirror. This mirror rotates, allowing the radiation measurement along a horizontal line or line-scan. Thus, at every turn, a measurement along a linear fringe over the object is taken, resulting in a line-scan [55]. The repeated line-scan measurement and the movement of the object forward along a track make the acquisition of temperature-discrete values over the whole surface of the object possible. Another approach is to use an array of detectors called a focal plane array. Each detector provides information about the radiation at one point.

An infrared image can be described as a function *f*(*x*, *y*). The amplitude value of *f* at spatial coordinates (*x*, *y*) is a scalar positive value, which corresponds to the amount of energy radiated from a region of a plane. The function *f* is continuous in space and amplitude. Thus, in order to convert this function to a digital format, it must be sampled in both space and amplitude. The digitization of the image space is called sampling, while the digitization of the amplitude is called quantization.

Sampling is defined by the resolution of the infrared camera, *i.e.*, the number of detectors of the FPA. The resolution describes the amount of information the camera can acquire, and it is indicated by two parameters: *M* and *N*, usually of the form *M* × *N*, the number of rows by the number of columns. Typical values span in the range 120 × 140 – 1280 × 1024.

Quantization is defined by the number of bits used to represent a discrete value of measured radiation. In order to carry out the quantization process, it is necessary to define the set of levels the signal can take. The signal range is then divided into that number of levels, and the continuous value is assigned to the level in which it is located. A typical value is 14 bits, *i.e.*, 16,384 levels. Quantization is also affected by the sensitivity, that is, the minimum temperature difference at two points of the image. This is commonly expressed as the noise-equivalent temperature difference (NETD). Typical values are around 20–50 mK.

A third aspect of digitization is temporal sampling, that is, the number of images or frames an infrared camera can acquire per second. Typical frame rates are 30 Hz and 60 Hz for full frames. However, using windowing, *i.e.*, acquiring only from a region of the frame, the frame rate can be increased significantly, over 30 kHz is some cameras.

## 3. Temperature Measurement

Temperature is one of the most frequently measured physical quantities. Temperature measurement provides information about the object internal energy, so its regulation and control is of vital importance in many industrial processes [56].

Temperature measurement using IRT measures the infrared radiation emitted by an object and converts the energy detected into a temperature value. However, as not all of the radiation received comes from the target object, to measure temperature accurately, radiation from other sources (such as surrounding objects or the atmosphere) must be removed in the conversion to temperature. This process is called compensation.

**Figure 5.**
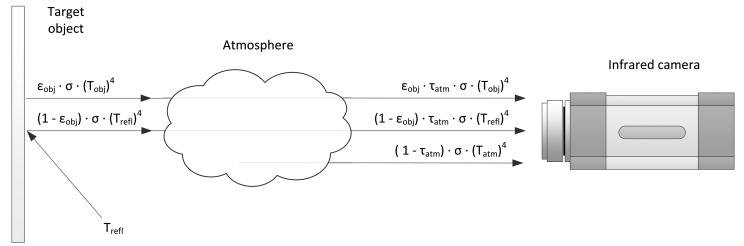
Radiation received by the infrared camera.

The total radiation received by the camera (*W_tot_*) comes from three sources: the emission of the target object (*E_obj_*), the emission of the surroundings and reflected by the object (*E_refl_*) and the emission of the atmosphere (*E_atm_*). It can be expressed as Equation (11). The process is illustrated in Figure 5.

(11) $$W_{\text{tot}}=E_{\text{obj}}+E_{\text{refl}}+E_{\text{atm}}$$

The first source is the emission from the target object. However, not all radiation emitted by the target object is received by the camera; as a function of the transmittance of the atmosphere (*τ_atm_*), some is absorbed by the atmosphere. Thus, the emission of the target object can be expressed as Equation (12).

(12) $$E_{\text{obj}}=\varepsilon_{\text{obj}}\;\cdot \;\tau_{\text{atm}}\;\cdot \;\sigma \cdot \;{\left(T_{\text{obj}}\right)}^{4}$$

Greybodies have a reflectivity greater than zero. Thus, they reflect the infrared radiation emitted by the surroundings. The reflectivity can be calculated from the emissivity, as shown in Equation (10). Part of this reflected radiation is also absorbed by the atmosphere. This is the second component received by the camera and can be expressed as Equation (13).

(13) $$E_{\text{refl}}=\rho_{\text{obj}}\;\cdot \;\tau_{\text{atm}}\;\cdot \;\sigma \;\cdot \;{\left(T_{\text{refl}}\right)}^{4}=(1-\varepsilon_{\text{obj}})\;\cdot \tau_{\text{atm}}\;\cdot \;\sigma \;\cdot \;{\left(T_{\text{refl}}\right)}^{4}$$

The third component is the emission of infrared radiation from the atmosphere. This can be expressed as Equation (14), where (1 − *τ_atm_*) is the emittance of the atmosphere.

(14) $$E_{\text{atm}}=\varepsilon_{\text{atm}}\;\cdot \;\sigma \;\cdot \;{\left(T_{\text{atm}}\right)}^{4}=(1-\tau_{\text{atm}})\;\cdot \;\sigma \;\cdot \;{\left(T_{\text{atm}}\right)}^{4}$$

Substituting Equations (12)–(14) in (11), Equation (15) is obtained. Therefore, the temperature of the object can be calculated from Equation (16). Similar equations are used by different camera manufactures to perform temperature measurements [57,58].

(15) $$W_{\text{tot}}=\varepsilon_{\text{obj}}.\tau_{\text{atm}}.\sigma .{\left(T_{\text{obj}}\right)}^{4}+\left(1-\varepsilon_{\text{obj}}\right).\tau_{\text{atm}}.\sigma .{\left(T_{\text{refl}}\right)}^{4}+\left(1-\tau_{\text{atm}}\right).\sigma .{\left(T_{\text{atm}}\right)}^{4}$$

(16) $$T_{\text{obj}}=\sqrt[{4}]{\frac{W_{\text{tot}}-\left(1-\varepsilon_{\text{obj}}\right).\tau_{\text{atm}}.\sigma .{\left(T_{\text{refl}}\right)}^{4}-\left(1-\tau_{\text{atm}}\right).\sigma .{\left(T_{\text{atm}}\right)}^{4}}{\varepsilon_{\text{obj}}.\tau_{\text{atm}}.\sigma }}$$

In order to solve Equation (16), the following parameters must be supplied: the emissivity of the object (*ε_obj_*), the reflected temperature (*T_refl_*), the transmittance of the atmosphere (*τ_atm_*) and the temperature of the atmosphere (*T_atm_*).

The transmittance of the atmosphere is generally estimated using the distance from the object to the camera and the relative humidity. In general, this value is very close to one. The temperature of the atmosphere is obtained using a common thermometer. However, as the emittance of the atmosphere is very close to zero (1 – *τ_atm_*), this parameter has little influence on the temperature measurement.

On the other hand, the emissivity of the object and the reflected temperature have a very high influence on the temperature measurement and must be measured very accurately.

### 3.1. Emissivity and Reflected Temperature Measurement

The most important calibration parameter for temperature measurement using IRT is emissivity. This parameter indicates how much radiation is emitted from the target object compared to that from a blackbody at the same temperature. Therefore, low-emissivity materials emit less infrared radiation than materials with high emissivity at the same temperature.

Accurate emissivity measurement is particularly important in low-emissivity materials. In objects with high emissivity, slight variations in the chosen emissivity value cause only minor changes in the resulting surface temperatures. However, in low-emissivity objects, such as polished steel or aluminum, temperature measurement is particularly complicated, because small variations in emissivity lead to large variations in the resulting temperatures.

**Figure 6.**
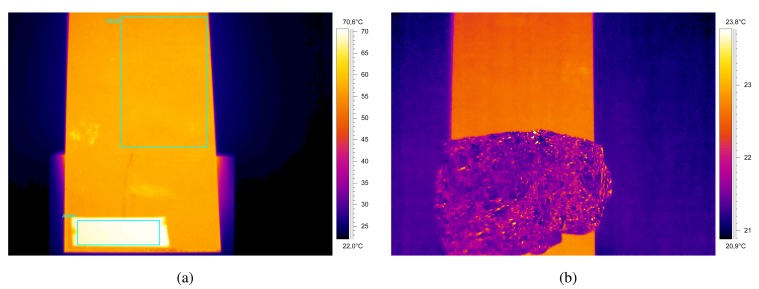
Infrared images acquired during calibration. (**a**) Measurement of the emissivity using the reference emissivity material method. (**b**) Measurement of the reflected temperature using the reflector method.

There is a general procedure for emissivity measurement [59,60]. The target object must be heated until it reaches the temperatures that will be reached under real working conditions. A thermocouple can be used to obtain a reference temperature, although for low temperatures, it is more common to use the same infrared device to be used in the temperature measurement, sticking a piece of electrical tape with known emissivity on the sample. The former method is called the contact method and the latter is called the reference emissivity material method. Once the real temperature of the heated piece is known, the sample is measured again with the infrared device, but this time on the surface of the object rather than on the electrical tape. The configuration of the emissivity is then changed until the real temperature is measured. The final configured emissivity is the emissivity of object. Figure 6a shows an infrared image acquired during the measurement using this method. As can be seen, the tape has very high emissivity, which results in a higher apparent temperature.

The reflected temperature is also an important parameter, especially when the reflectivity of the target object is high (low-emissivity materials). There are two common methods to measure the reflected temperature: the reflector method, and the direct method [60,61]. The reflector method is much more common, as it is easier to apply and provides better results. The reflector method uses a calibrated reflected standard, such as gold metallic coating (with 95% constant reflectivity from 2 to 20 μm). A commonly used alternative is a crumpled and re-flattened piece of aluminum foil. The reflector is placed in the field-of-view of the infrared camera, and the temperature of the reflector is measured assuming an emissivity of one and a distance of zero. Finally, the measurement is repeated by using the temperature of the reflector as the reflected temperature. The resulting temperature value is the final reflected temperature. Figure 6b shows an infrared image acquired during the measurement using this method.

### 3.2. Research on Temperature Measurement Using IRT

Research on temperature measurement applications based on IRT is mainly focused on the experimental setup. The most adequate configuration is application dependent and greatly influences the accuracy of the results.

There is a general procedure for emissivity measurement. However, there are cases where the general procedure is not applicable, such as in small-sized, distant objects [62]. In other cases, such as in industrial environments, emissivity is not known accurately [63], as it changes during the production process. Therefore, some works have proposed alternative methods for emissivity measurement. Techniques based on multi-wavelength pyrometers are widely used approaches to solve this problem [64,65]. However, these techniques are based on the assumption that a simple relationship between wavelength and emissivity exists [1]. In [66], an application of multi-wavelength pyrometers is presented. In this case, three wavelengths are taken, and the emissivity of the object is approximated with constant, power and exponential functions. The proposed method is especially useful for measurements of metal surfaces, because the approximation function for their spectral emissivity is a power function. The method also ensures minimal measurement error. In [67], a methodology is presented for temperature measurement in the cutting process. This work analyzes the advantages of multi-wavelength pyrometers, and it concludes that two-color thermometry is not the most suitable method for temperature estimation on the tool insert. The reason is that the assumption required to apply multi-wavelength measurements of the temperature without knowledge about the emissivity is not true. This work also includes a complete analysis of the measurement of the emissivity. In a previous work, the authors measured the emissivity in different working conditions using a high-precision radiometer [68]. Different variables, such as the influence of temperature and wavelength, the influence of roughness and the influence of oxidation, are considered. This work confirms that, based on these variables, an incorrect measurement of emissivity produces important temperature shifts.

**Figure 7.**
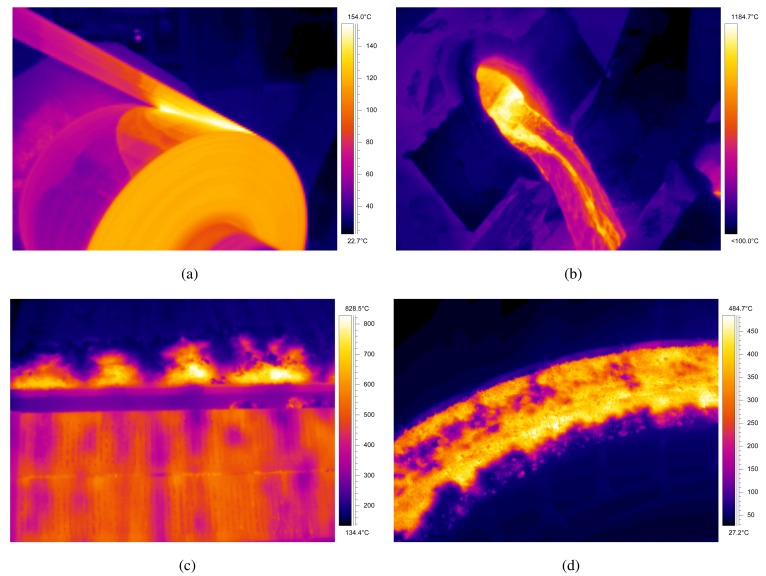
Temperature measurement in different applications. (**a**) Temperature measurement in steel strips using the wedge method; (**b**) Temperature measurement of pig iron; (**c**) Temperature measurement in sinter material; (**d**) Temperature measurement in a rotatory cooler.

One effective method to overcome the problem of measuring the emissivity is to measure temperature where the material forms a wedge that creates a virtually closed cavity for radiation (Figure 7a). In the deepest part of the wedge, the cavity acts as a virtual blackbody, where emissivity is very stable and close to one. Temperature measurement using this method, which is often called the wedge method [69], is much more precise. In [70,71], the wedge method is used to measure the temperature of polished steel strips with low emissivity, around 0.37. These works use the wedge that is created in the strip coil. The region is detected, and the measurement is performed in this region, with an improvement of 6%.

The wedge method is used to increase emissivity. However, it is only possible when the material forms a cavity. Another option is to create an artificial cavity in the material by drilling a hole. The hole must be at least six times deeper than its diameter. This will produce an effective emissivity close to one [72]. This effect can also be simulated by surrounding the material with a high-reflectance enclosure. The disadvantage of this approach is that it requires contact with the material, interfering with the measurement and making it unsuitable for continuous processes.

The problem with low-emissivity objects is that slight variations in the chosen emissivity value lead to large variations in the resulting temperatures. Thus, it is better to increase emissivity during the experimental setup. A simple way to enhance emissivity is to paint the material. High emissivity paint permits accurate temperature measurement in almost any background conditions [73]. The increased accuracy is related to the increased emissivity of the surface and to the fact that the emissivity of the paint is a known value. Measuring the temperature over the raw low emissivity surface requires an estimation of an unknown value: the real emissivity of the object. Moreover, a slight variation of the estimated emissivity (low value) of the object will lead to large variations in the resulting temperature reading. In addition, the temperature measurement of high emissivity objects is not affected by reflections. Thus, the conditions of the environment are much less important when the material has been painted.

In other applications, emissivity is not an issue, because it is known accurately; however, there are other variables that greatly influence the measurement. This is the case of temperature measurement in clinical practice [74], where the emissivity value for skin temperature is known [75]. The place where temperature measurement is carried out must be a room at controlled homogeneous temperature and free from any secondary infrared sources, such as lamps. The subjects require an acclimation time in the room to achieve thermal equilibrium. They must rest during this time in a comfortable position. Prior to the measurements, subjects must follow some instructions, such as no sunbathing and no use of lotions or creams. Patients also must abstain from consuming alcohol or caffeine for a 4-h period prior to the start of the procedure. Many different applications can be found where these procedures are followed. In [76], IRT is used as a non-invasive assessment tool to detect inflammation for acute, as well as chronic care for the foot. In this case, IRT is used to prevent diabetic foot complications, thus, preventing a serious health problem that can lead to major amputations. In [24], the approach used is different: rather than for prevention, IRT is used to assess the evolution of a medical treatment, in particular for leprosy and hepatitis C. The results indicate that IRT was able to detect the effectiveness of the treatment in spite of variable environmental conditions. A similar approach is used in [77]. In this case, low-intensity laser irradiation is applied to patients with diabetic microangiopathy to improve blood circulation. The improvement of skin blood circulation is controlled using IRT. Again, IRT was very effective in assessing the evolution of a medical treatment.

Another problem in medical applications is how to obtain a single temperature value from the images. An infrared camera produces an infrared image with thousands of points where temperature has been measured. However, medical practice requires a single temperature reading. Thus, a procedure is required to combine the available information. In [78], different strategies are analyzed: Tmax calculates an average of the temperature values of all of the pixels within the region considered; Troi calculates an average of the area of 5 × 5 pixels around the hottest pixels; and Ttot calculates an average of the temperature values of all of the pixels included in the upper part of the temperature distribution in the region of study. The authors conclude that these methods provide very similar results. However, Tmax is considered the most useful, as it can be used for both non-static shooting and when a fast thermal response is considered.

Some works have focused on detecting the region of interest (ROI) in the infrared images. In most applications, infrared images contain information about the target object, but also about the surroundings. Thus, in order to determine the temperature of the target object, it needs to be identified in the image. This region of the image is usually called the ROI, *i.e.*, the portion of an image of particular interest. The ROI is application dependent. In [79] (Figure 7b), the ROI is a stream of pig iron. The procedure followed in this work is an image processing algorithm based on the following steps: detection of the open mouth using edge detection and fitting, definition of an ROI around the mouth of the torpedo and segmentation using thresholding and region growing. In [80], the ROI is the flame front in a sinterization process (Figure 7c). In this case, the procedure segments the ROI using active contours. In [81], the ROI is a rotatory cooler (Figure 7d). In this case, the ROI is detected using a combination of edge detection and fitting. The movement of the hot material is also tracked by estimating the geometric transformation between images.

In some applications, it is necessary to extract spatial information from infrared images, *i.e.*, the world position associated with each pixel must be known. This issue has been widely dealt with in the image processing field applied to traditional cameras [82,83]. In IRT, spatial calibration is not common; however, there are specific calibration procedures, both for infrared line-scanners and for infrared cameras. In [84], a procedure is proposed for the calibration of infrared line-scanners, and the uncertainty is analyzed. In this case, the calibration is based on a specific model of the infrared line-scanner used. In [85], a procedure is proposed for the calibration of infrared cameras. It is based on a grid of burning lamps that is used for the geometric calibration. This device is used to create correspondences between logical coordinates (sensor) and physical coordinates (world). The authors apply the proposed procedure to two different infrared cameras and achieve an accuracy lower than 1 mm. In [86], a procedure is proposed for the calibration of infrared cameras using a different approach. The work explores the use of small lamps that warm up when switched on, but concludes that this approach cannot be used to obtain high accuracy. Instead, the authors propose a new calibration device based on the reflectance of metals from the cold temperatures of outer space. The results provide an accuracy lower than 0.25 mm for common infrared cameras. In general, these works use calibration devices to build a model that can be used to determine the relation between camera units (pixels) and real world units (millimeters). This gives a benefit to the temperature measurements, as spatial information can be added to these measurements.

## 4. Non-Destructive Testing

Infrared thermography applied to non-destructive testing (NDT) measures and interprets the temperature field of the surface of the body being studied. The theoretical principle is based on the fact that the internal structure of the inspected object and its flaws will have a different thermal behavior. The defects affect the flow of a previously applied heat source, which will be heated or cooled at different rates. The result is temperature differences on the surface of the object (thermal contrast), resulting from differences in radiation emission captured by the infrared camera. Data processing techniques are applied to the acquired data when the acquired signal is weak to improve the results, making defect detection possible.

NDT-based on IRT can be applied using a passive or an active approach. In the passive approach, the material being inspected needs to be at a different temperature. This approach is used, for example, in the inspection of aircraft structures to discover water inside panels. The active approach is much more common. In active thermography, acquisition is carried out at the same time as an external stimulus is being applied to the inspected specimen. The objective is to create a thermal contrast on subsurface anomalies, *i.e.*, the defects. Many different stimulation methods can be applied. Most of them can be classified as optical, mechanical or inductive. The most common are based on optical stimulation, which uses light to deliver energy to the specimen. The applied stimulation generates heat, which propagates as thermal waves from the surface through the specimen. When the thermal waves reach an anomaly, they change their propagation rate, producing a thermal contrast on the surface immediately above the anomaly [87].

**Figure 8.**
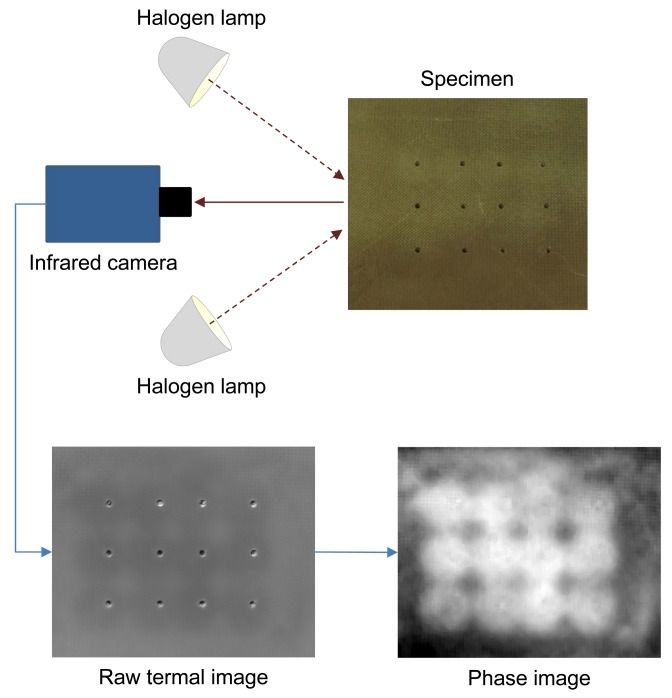
Schematic setup of a possible active thermographic inspection.

Figure 8 shows a possible configuration for NDT using active thermography. In this example, the halogen lamps are used to apply a step heating (long pulse) to the specimen, while its surface temperature is monitored as a function of time. Both the temperature increase and decay are of interest. This particular configuration was used to inspect drilled holes in reinforced honeycomb sandwich panels using active thermography in [88]. Some subsurface anomalies are so subtle that the signal levels associated with them can be lost in the thermographic data noise. In these cases, the acquired raw thermographic data does not provide very useful information. One possible solution is to apply a post-processing method in order to improve the SNR content of thermographic data. In Figure 8, the result of one of these post-processing methods is shown: the phase of the discrete Fourier transform (DFT). As can be seen, the resulting image provides information about the reinforced areas that were invisible to the human eye.

In general, three elements are involved in NDT using IRT: an infrared camera, an excitation source and a data processing algorithm to improve the SNR [89]. These elements have experienced a great evolution in a relatively short time since the beginning of this technology.

### 4.1. Infrared Cameras for NDT

Depending on the characteristics of the object to be measured and the environmental conditions, a specific infrared camera may be more suitable for NDT inspection. The selection of the spectral band essentially depends on the spectral emissivity of the materials, the radiation from the object, the thermal contrast, the atmospheric transmittance, the type of detector and the parasitic radiations. However, the influence of each of these parameters is rather difficult to evaluate individually or globally. For example, an LWIR camera is less influenced by the presence of moisture in the atmosphere than an MWIR camera. However, an MWIR camera is less influenced by optical and electronic noise. The emissivity of the object to be measured is another important parameter to be taken into account for the election of an infrared sensor. Bearing in mind that the emissivity is a wavelength-dependent parameter, the higher the emissivity, the less the parasitic reflections and, therefore, the better the detection. A recent review of infrared sensors for NDT can be found in [90], where the performance of MWIR and LWIR cameras are compared.

### 4.2. Excitation Sources

**Figure 9.**
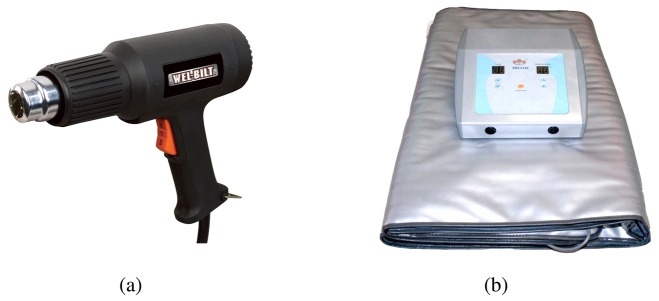
First stimulation tools used in non-destructive testing (NDT) with infrared thermography (IRT). (**a**) Heat gun. (**b**) Thermal blanket.

The application of IRT as an NDT technique requires an extra heat supply to induce a controlled change in its temperature. The first stimulation systems that were widely employed for NDT inspections using IRT were thermal blankets and heat guns [91–93]. These tools can be seen in Figure 9.

Thermal blankets provide precise heating of regular surfaces of medium-and large-sized objects, and they can usually be programmed for specific heating cycles for different types of materials. The drawback of using thermal blankets is that it is only possible to analyze the cooling-down stage, because during the warming-up stage, the blanket covers the object, preventing data acquisition. The heat gun is one of the most extended stimulation methods, due to its simplicity, low cost and flexibility. It warms up the object using hot air directly oriented towards the point of interest. This is a very quick stimulation method suitable for medium-and small-sized surfaces, depending on the size of the gun. The main drawback of this technique is the low repeatability rate, since it is usually applied manually.

**Figure 10.**
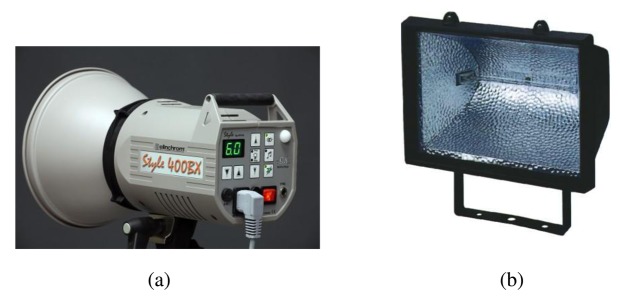
Common optical stimulation sources in NDT. (**a**) Flash lamp. (**b**) Halogen lamp.

Thermal blankets and heat guns are the simplest stimulation techniques that were initially used for NDT inspections by IRT. They are still in use nowadays, providing excellent results in specific applications. However, to overcome the drawbacks of these techniques and to improve the defect detection rate, new excitation techniques have been developed. The most popular are the well-known optical techniques [94,95]. They are applied by means of flash lamps, which emit pulses of energy; and halogen lamps, for both continuous and modulated heating. Both technologies can be used to stimulate large or small areas by changing the number of lamps, analyzing not only the cooling-down, but also the warming-up stage, and providing a high repeatability rate. Figure 10 shows some of the most common lamps used in optical stimulation. For large spaces, gas blow heaters that use propane or diesel fuel are also commonly used.

In general, optical stimulation techniques provide very good results. However, there are specific defects, such as cracks in metallic parts and some inserts in carbon-fiber-reinforced polymer (CFRP) materials, in which optical stimulation techniques do not provide enough thermal contrast. To overcome this limitation, advanced stimulation techniques were developed, including vibrothermography and thermoinduction thermography [96–99].

In vibrothermography, the heat generated by the friction of the discontinuities, cracks or even delaminations is induced by the effect of mechanical excitation (20–50 Hz) applied externally to the structure. These discontinuities are excited under specific mechanical resonances. Depending on the variation of the frequency of mechanical excitation, the local thermal gradients that indicate the presence of the defect can appear or disappear.

Ultrasound thermography (UT) is a variation of vibrothermography. While the structure under study is vibrated in vibrothermography, in UT, a piezoelectric effectintroduces ultrasonic waves that propagate through the material. A high-frequency ultrasound signal is generated at 40 kHz and is additionally modulated with another lower frequency signal. The test configuration is as follows: a horn (sonotrode) injects ultrasound waves into the material; low frequency waves make spreading possible, while high frequency vibration produces heat by the friction of particles.

**Figure 11.**
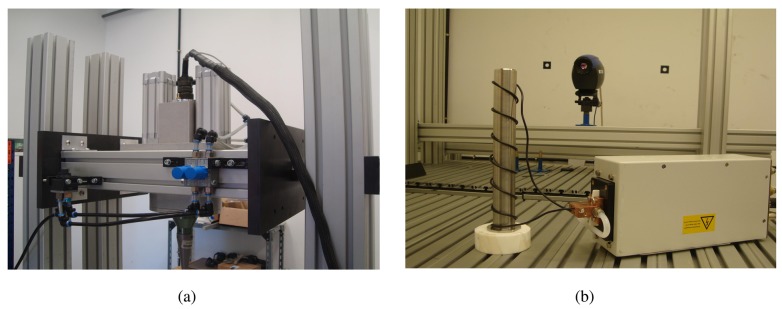
Ultrasound and induction stimulation techniques for NDT inspection using IRT. (**a**) Ultrasound gun. (**b**) Induction coil.

Thermoinduction thermography creates eddy currents within the material to be inspected by circulating a current at certain frequencies along an induction coil. The current density where the defects are located is different, producing heat on the surface. Figure 11 shows the tools used in ultrasound and induction stimulation techniques.

New research is being carried out to develop advanced stimulation methods adapted to specific types of defects with very promising results. This is the case of laser excited thermography [100], microwave excited thermography [101] and the focalized optical thermography [102].

In addition to the excitation technologies described above, in NDT using IRT, the technique employed for generating the necessary heat is also of great interest. Depending on how the heat is introduced into the structures, different methods are differentiated in thermography, resulting in:

- Conventional thermography or step heating (continuous heating).
- Pulsed thermography (short heating, pulse of heat).
- Lock-in or modulated thermography (cyclic modulated heating). In this case, it is necessary to synchronize heating and acquisition.

Pulsed thermography involves briefly heating the specimen with a short pulse of thermal stimulation and then recording the temperature decay curve [103–105]. The temperature of the material varies rapidly after the initial thermal pulse, while the thermal front propagates by diffusion through the surface. The presence of a discontinuity reduces the diffusion rate, so that, by observing the temperature of the surface, the discontinuities appear as areas of different temperatures with respect to the surrounding sound areas. Therefore, deeper discontinuities will be observed later and with a smaller contrast.

In lock-in or modulated thermography, the surface temperature field is also studied, but in this case, thermal waves have been created inside the object rather than a thermal front [106,107]. The temperature of each point on the surface will vary over time, as it will be affected by the generated waves and those reflected in thermal barriers, for example, defects. These temperatures are recorded by an infrared camera that is synchronized with the excitation signal. Each pixel in the thermal image corresponds to a temperature at a given time, so that by the application of some specific calculations for each pixel per cycle, an improved result is obtained. This new result is usually expressed as an “amplitude image” or “phase image”, which is less affected by heating heterogeneities, the presence of noise, external reflections, emissivity variations and, in general, is more sensitive than the original thermograms. The different thermal barriers inside the body will be reflected in these images, so we can see the internal structure of the body and all possible defects. While it is more sensitive, this technique can be very slow, as for each depth to be inspected within the material, a determined test at a given excitation frequency must be conducted. Moreover, each of these tests requires several cycles of excitation to achieve a steady state.

In step heating thermography, the structure to be studied is continuously heated or cooled, and its evolution is observed [108]. Unusual behavior in the evolution curves of an area, either in heating or cooling, determines the existence of an irregularity or defect. That is, the defects will be indicated as areas heated or cooled at different rates, appearing as hot or cold spots.

Each type of defect in a specific material offers a different response to the application of the different IRT NDT techniques. It is important to find the most suitable technique (deposited energy, distance between heat sources and target object, heating times, and so on) for the detection of each type of defect and the parameters that characterize each one (time of detection, highest contrast).

### 4.3. Data Processing

More recently, after the development of advanced excitation technologies, a new research line began to gain great importance. This new line deals with data processing algorithms, which are used not only to improve the level of detection of the IRT technology, but also to characterize the detected defects in order to automate the inspection process [109]. Some of the most important data processing techniques used in IRT are statistical moments, principal components analysis, dynamic thermal tomography, polynomial fit and derivatives and pulsed phase thermography.

#### 4.3.1. Statistical Moments

Data obtained with infrared thermography is a sequence of numerical values. As a consequence, these numerical values could be treated by statistical functions describing certain behaviors and to detect significant changes between some values and others. For this reason, different statistical moments, offering different results, are used.

Skewness is the third standardized statistical moment from a distribution. The term moment is used to represent the expected values of the different powers from a random variable [110]. It is used to determine the degree to which data fits a given type of distribution. The mathematical formula used to calculate skewness can be seen in Equation (17), where μ is the mean value and σ is the standard deviation of the random variable x. *E* is the mathematical expectancy, defined as Equation (18), where *n* is the number of data points.

(17) $$k_{3}=\frac{E\left[{\left(x-\mu \right)}^{3}\right]}{\sigma^{3}}$$

(18) $$E\left[X\right]=\frac{1}{n-1}\sum_{i=1}^{n}x_{i}$$

Using skewness, it is possible to measure the asymmetry of the probability distribution from a random variable of real parameters. This method is an appropriate processing technique for infrared images, and it was found that the application of this statistical method is hardly affected by non-uniform heating or by the shape of the surface of the tested material [111].

Kurtosis is the fourth standardized statistical moment from a distribution. It is generally defined as a measure that reflects the degree to which a distribution has a peak shape [112]. In particular, kurtosis provides information about the height of the distribution in relation to the value of the standard deviation. Mathematically, it is defined as Equation (19).

(19) $$k_{4}=\frac{E\left[{\left(x-\mu \right)}^{4}\right]}{\sigma^{4}}$$

The temperature distribution of a defect in an image has a kurtosis value different from an area without defects, depending on the thermal diffusivity of the defect area. Therefore, it is possible to estimate the kurtosis values for every pixel in the image sequence and to obtain a unique image showing these values: a kurtogram. The kurtogram gives an indication of the location of defects on the subsurface and their thermal diffusion [113].

#### 4.3.2. Principal Component Analysis

Principal component analysis, or PCA, is a statistical technique of information synthesis. Its objectives are, firstly, to reduce the number of variables in a data set to a smaller number, losing the least amount of information possible, and, secondly, to highlight the differences and similarities in data.

In [114], the steps for applying this method to a set of data are described, and in [115], the effectiveness of this method for reducing thermographic data is shown.

Processing based on PCA uses a set of statistic orthogonal functions, known as empirical orthogonal functions (EOF), to decompose the thermal sequence of the surface temperature variation of a specimen obtained after a pulsed active thermography test into its principal components. In this way, data can be reduced without deleting useful information. These principal components are obtained from the singular value decomposition (SVD) of the thermic temporary data matrices [116]. The method is called principal component thermography (PCT).

#### 4.3.3. Dynamic Thermal Tomography

Dynamic thermal tomography (DTT) means “layering” a test specimen in different layers corresponding to different depths, in which the distribution of the thermal properties can be observed. This technique is based on the analysis of the surface temperature evolution after applying an external thermal excitation [117].

This algorithm is applied to a thermal image sequence where the evolution in time of the temperature is observed. In order to utilize this technique, the time evolution of each pixel in the image is fit through different order polynomials. While the low order polynomials describe the behavior of the areas without defects, higher order polynomials describe the variations of the defects. Therefore, the differential for each pixel is expressed as Equation (20):

(20) ΔT(i,j,τ)=Th(i,j,τ)−Tl(i,j,τ)

The DTT algorithm returns two different images as a result: the maxigram, where the maximum values of Δ*T* are observed, and the timegram, which indicates the time at which these maximum values take place.

#### 4.3.4. Pulsed Phase Thermography

Pulsed phase thermography (PPT) is based on the phase calculation of a sequence of images, in which the time history of each pixel describes the thermal propagation of an external energy excitation [118,119]. In order to apply this method, PPT transforms the image sequence to the frequency domain using the DFT, as seen in Equation (21), where *i* is the imaginary number, *n* is the frequency increment and *Re_n_* and *Im_n_* are the real and imaginary parts of the DFT.

(21) $$F_{n}=\sum_{k=1}^{N-1}T(k)\;e^{\frac{2\pi \text{ikn}}{N}}={\text{Re}}_{n}+{\text{Im}}_{n}$$

Finally, the phase of each pixel is defined as Equation (22):

(22) $$\emptyset_{n}=\text{atan}\left(\frac{{\text{Im}}_{n}}{{\text{Re}}_{n}}\right)$$

This technique combines the advantages of modulated and pulse infrared thermography [95].

### 4.4. Polynomial Fit and Derivatives

This technique is based on the polynomial fit of each pixel time history from a given thermographic image sequence. This method is often called thermographic signal reconstruction (TSR) [120]. The evolution of the temperature is adjusted to an n degree polynomial, as shown in Equation (23):

(23) $$T\left(t\right)=a_{n}t^{n}+a_{n-1}t^{n-1}+\ldots +a_{1}t+a_{0}$$

This method enhances and filters thermographic data, reducing the noise content. It can also be used as a previous step for other types of processing techniques. In order to improve the results, a logarithmic conversion of the time history of each pixel is calculated using Equations (24) and (25):

(24) $$\ln\left[T\left(t\right)\right]=a_{n}{\left\{\ln\left[t\right]\right\}}^{n}+a_{n-1}{\left\{\ln\left[t\right]\right\}}^{n-1}+\ldots +a_{1}\left\{\ln\left[t\right]\right\}+a_{0}$$

(25) $$T\left(t\right)=\text{exp}\left(a_{n}{\left\{\ln\left[t\right]\right\}}^{n}+a_{n-1}{\left\{\ln\left[t\right]\right\}}^{n-1}+\ldots +a_{1}\left\{\ln\left[t\right]\right\}+a_{0}\right)$$

In these logarithmic curves, it is possible to observe the thermal propagation difference between a pixel with a defect and a defect-free pixel. Second and third derivatives of the polynomials offer more information about defects.

### 4.5. Comparison of Data Processing Techniques

**Figure 12.**
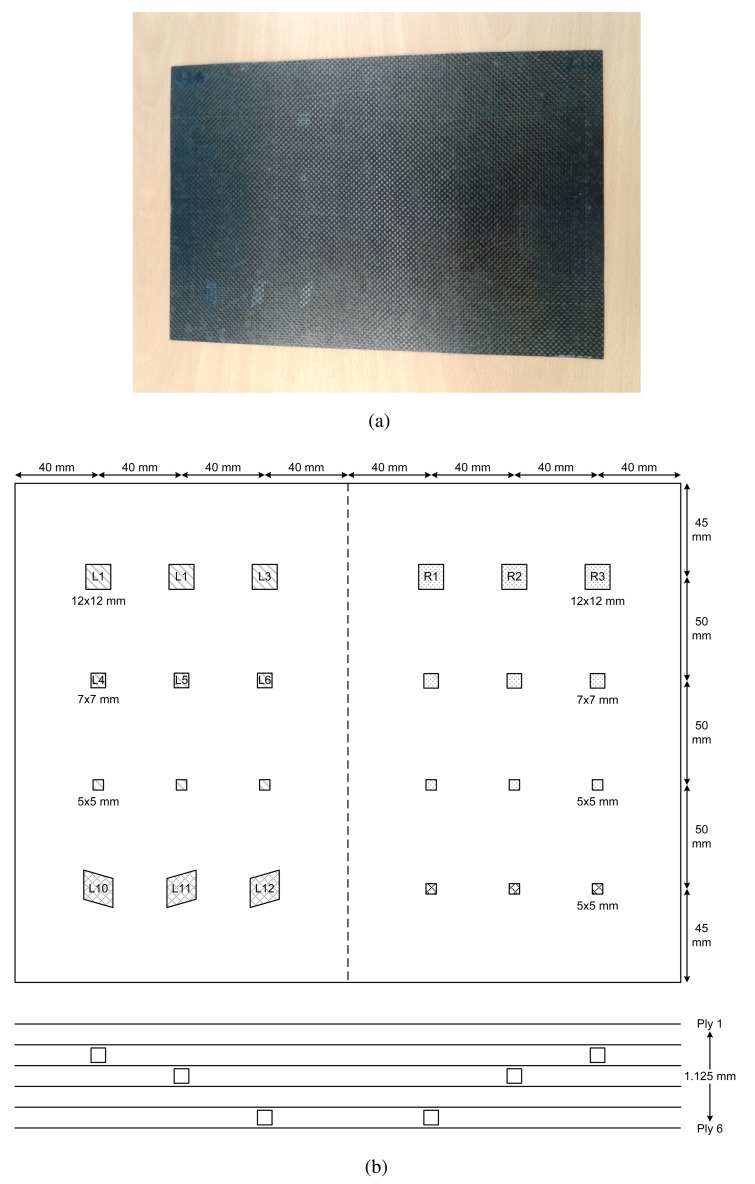
Specimen under study. **(a)** Photo of the specimen; **(b)** Map of the defects.

In order to compare the reviewed data processing techniques, they have been applied to a real piece with defects. Figure 12a shows the test piece used in the experiments: it is a CFRP specimen used in the construction of aeronautical structures. The test piece employed in the tests has several artificial defects of different types, sizes and depths. The map of defects can be seen in Figure 12b.

The specimen is made of several layers of carbon-fiber filled with epoxy. Each layer is numbered from 1 to 6, where Layer #1 is the layer closest to the surface on which the temperature is measured. The total thickness is 1.125 mm. The specimen is divided into two zones: left and right. In the left zone, there are 12 defects numbered from L1 to L12, from left to right and from top to bottom. Defects L1–L9 are remnants of film attached to a carbon-fiber layer (filled with horizontal lines in the map). Defects L10–L12, on the left side, are remnants of metal attached to a carbon-fiber layer (filled with points in the map). On the right side of the specimen, there are another 12 defects, R1–R12. Defects R1–R9, on the right side, are remnants of Teflon attached to a carbon-fiber layer (unfilled). Defects R10–R12, on the right side, are remnants of metal. As can be seen in the map, these defects are located at different depths; some are under two layers, others under three and others under five layers. As can be appreciated in Figure 12a, some defects can be seen in the photo, because the test piece is very thin, while most of them are invisible to the human eye.

In the experiments, optical step heating stimulation was used with three 1000 watt halogen lamps. The lamps were turned on for 10 s to heat the specimen. After the lamps were turned off, the temperature decay was recorded for another 15 s, making a total of 25 s. The warming-up and the cooling-down phases contain similar information. Thus, in this case, only the warming-up phase is considered.

All of the experiments were recorded using an FLIR SC5000 camera. This camera is equipped with a cooled Indium antimonide detector that operates in the 2.5 to 5.1 μm waveband. The FLIR SC5000 produces thermal images of 320 × 256 pixels with 12 bits per pixel and has a thermal sensitivity of 20 mK. Although the camera has a maximum frame rate of 383 Hz, the experiments were recorded at 50 Hz to reduce the number of acquired images. The field of view of the camera was 20 × 16 (27-mm lens built in), and the distance between the camera and specimen was 50 cm, while the distance between the lamps and the specimen was 60 cm.

The SNR metric is used to quantitatively assess the signal-to-noise ratio of the acquired and processed data [15]. The quantification of a defect is based on the definition of two areas: the defective area and the reference area. The defective area encloses the pixels where the defect appears in the infrared image. This area is considered the signal. The reference area, also called the sound area, is a defect-free area used to calculate the thermal contrast. This area is considered the noise. The reference area is selected independently for each defect and must be located close to it. Thus, the reference area is known to have received the same excitation energy as the defect, which minimizes errors in SNR calculations due to non-uniform heating.

The SNR metric for a defect is calculated as shown in Equation (26), where *Def_μ_* is the arithmetic mean of all of the pixels inside the defective area, *Ref_μ_* is the arithmetic mean of all of the pixels inside the reference area and *Ref_σ_* is the standard deviation of the pixels inside the reference area.

(26) $$\text{SNR}=20\log_{10}\left(\frac{\left|{\text{Def}}_{\mu }-{\text{Ref}}_{\mu }\right|}{{\text{Ref}}_{\sigma }}\right)$$

The results of the experiments can be seen in Tables 1 and 2, for the left and right side of the specimen, respectively. The figures use a color scale in which green indicates a good SNR, yellow is a medium SNR and red is a null SNR. The SNR was only calculated when the defect was recognizable in the resulting images. Thus, the null values in the table indicate that the method was unable to identify that defect.

**Figure 13.**
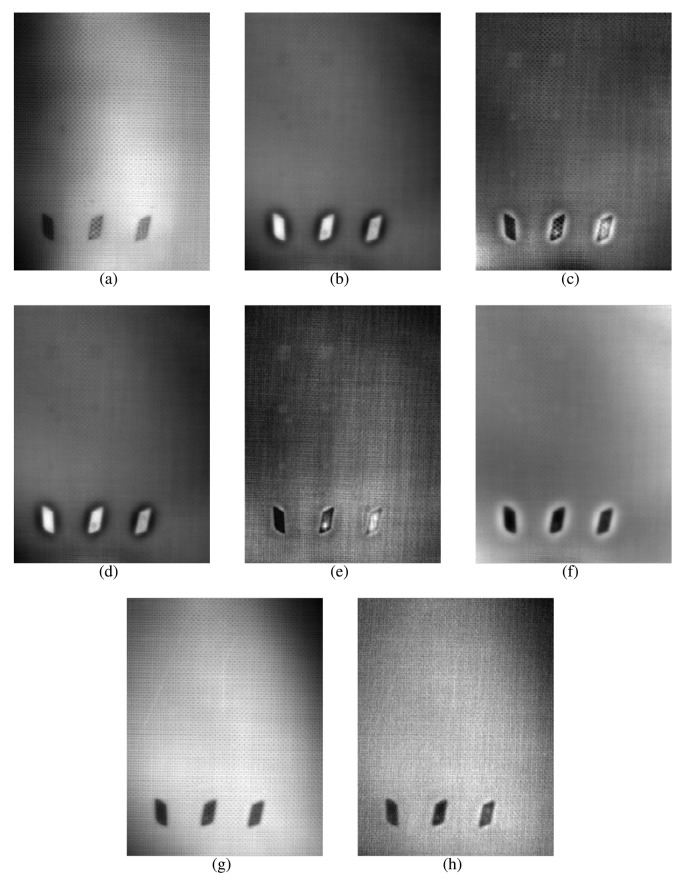
Resulting images from the experiments on the left side of the specimen. (**a**) Raw infrared image; (**b**) Skewness; (**c**) Kurtosis; (**d**) Principal component thermography (PCT); (**e**) Dynamic thermal tomography (DTT); (**f**) Phase image; (**g**) First derivative of the polynomial fit; (**h**) Second derivative of the polynomial fit.

**Figure 14.**
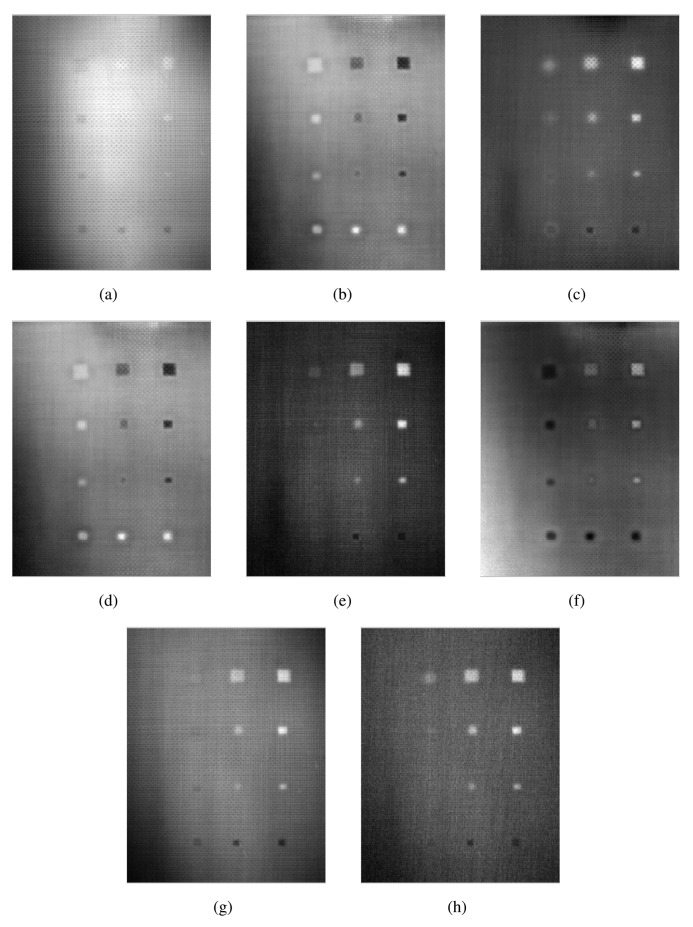
Resulting images from the experiments on the right side of the specimen. (**a**) Raw infrared image; (**b**) Skewness; (**c**) Kurtosis; (**d**) Principal component thermography (PCT); (**e**) Dynamic thermal tomography (DTT); (**f**) Phase image; (**g**) First derivative of the polynomial fit; (**h**) Second derivative of the polynomial fit.

The images resulting from the experiments can be seen in Figures 13 and 14, for the left and right side of the specimen, respectively.

The results of these experiments show a huge difference between the raw infrared image and the images resulting from applying data processing techniques. As can be seen in the first column of Table 2, the raw infrared images have a very low SNR. Most of the defects would go undetected using this information. On the other hand, data processing techniques increase the SNR significantly. Thus, these techniques are helpful in improving the SNR. These differences can also be seen in the images, especially in Figure 14. Another advantage of data processing techniques is that they tend to cancel part of the non-uniformities in the infrared images.

Results show a correlation between the depth of the defect and the SNR; in general, the deeper the defect, the lower the SNR. This is a known issue of non-destructive thermographic inspection, because the detection of deeper defects requires more energy. Defects R3, R6 and R9, located close to the surface between Layers #2 and #3, have a high SNR. However, deeper defects of the same type become more difficult to detect, such as R1, R4 and R7.

The diameter of the defect has a slight influence on the resulting SNR. In the case of R3 and R6, there is virtually no difference in terms of SNR. However, comparing Defect R6 with Defect R9, the average SNR is much higher for the bigger defect. The same applies to defects at other depths.

The type of defects also has a strong correlation with the SNR. The key to understanding this influence is the properties of the material, in this case of the remnants between layers. Thermal conductivity and density play an important role in the results. When the thermal conductivity is high, the resulting thermal contrast is also high. Thus, the SNR is very good. This is the case of defects with metal remnants. For example, Defects L10, L11 and L12 are clearly seen in the resulting thermographic images, even without using any post-processing methods. For these defects, the SNR is high enough not to need any additional data processing technique. The opposite happens with L1–L9. In this case, the properties of defects with remnants of film make them nearly invisible to the thermographic inspection. Not even data processing techniques can improve the SNR in these cases. The resulting images from PCT and DTT faintly show some of these defects in Table 1, but the SNR is too low, even in these cases. The problem with these defects is that the thermal diffusivity of the remnants of film is very similar to the thermal diffusivity of the CFRP used to build the test piece. Thus, the defects do not show a different thermal contrast than the material when they are stimulated.

Comparing data processing techniques is difficult, because there is no single method that maximizes the SNR for all materials. The most appropriate method depends on the properties of the sought anomalies. For the test specimen used in these experiments, PCT and the phase of the DFT seem to provide the best results on average. Skewness and kurtosis also provide good results, but they fail to identify some defects. However, there is no single data processing technique that maximizes SNR and the defect detection rate. The selection of a data processing technique and, in general, of the experimental setup depends on the particular application.

### 4.6. Applications

The application of IRT as an NDT tool has become widespread in recent years. Thanks to its particular features for rapid contactless inspections at a distance, IRT is currently a very flexible tool for NDT. Thermal measurement methods have a wide range of uses: from military applications for night vision and emergency rescue personnel, to the medical profession, industrial energy audits, preventive maintenance and process control.

The application of IRT as an NDT tool is suitable for many industrial sectors. Some significant examples of such IRT applications have been carried out in the automotive industry, where there is an ever-increasing demand for reliable, non-destructive testing methods. Mass production requires short cycle times and automated processes, so defect selective NDT-techniques are very appreciated for such applications. The main advantage of such “dark field” methods is the high probability of defect detection: they only show the defects. Ultrasound burst phase thermography (UBP) is one example of an established defect selective testing method [96]. In this work, ultrasound excited thermography (UET) is used as a stimulation source for crack detection in automotive components (aluminum pressure casting), characterization of shrink fits/press fits and characterization of adhesive-bonded joints.

UT thermography is a defect selective NDT technique that shows only the defects themselves. UBP is a defect selective imaging system using thermal waves generated by elastic waves. The mechanism involved is local friction or hysteresis that warms the defects. Thermography can then identify even complicated shapes. UBP combines the advantages of both lock-in and pulsed thermography: faster measurements with better reproducibility with depth resolved recognition of defects and suppression of inhomogeneous emissivity and temperature gradients.

A clear advantage over conventional optical pulsed IRT (OPT) is the removal of sensitivity variations within the detector, as well as optical sample characteristics, e.g., inhomogeneous temperature distribution and varying emissivity coefficients on the surface. Moreover, the SNR ratio is improved significantly, and consequently, the probability of defect identification is also improved. Furthermore, automation is possible, offering a broad spectrum of applications in the automotive industry.

**Figure 15.**
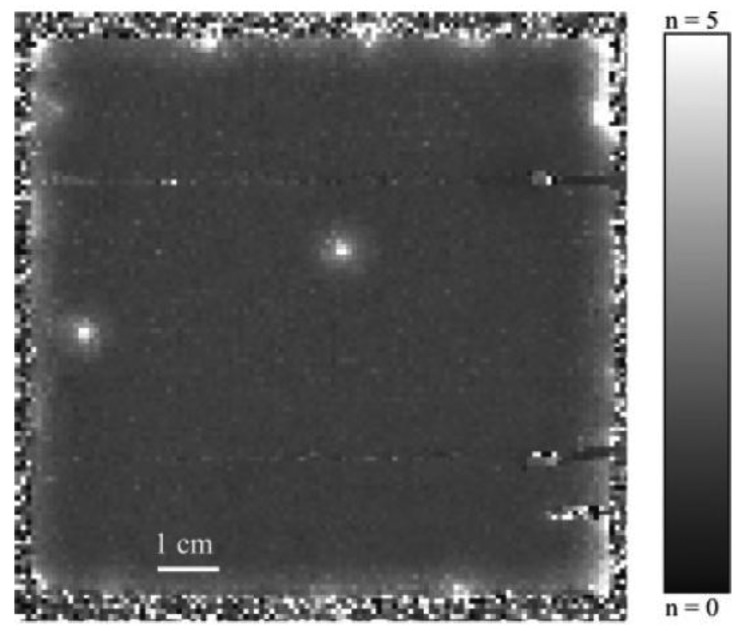
Lock-in thermogram (amplitude image, scaled to 2 mK) of the EFGsolar cell measured at 0.55 V (frame with permission from [121]).

Another industrial sector in which NDT by IRT is being increasingly applied is the renewable energy sector. Different fields of application can be found, e.g., wind turbines and solar panels. Recent studies [121] conclude that lock-in thermography allows a quantitative analysis of the spatial distribution of the dark forward current density of solar cells in order to detect possible failures and correct malfunctions. In Figure 15, a resulting thermogram of the lock-in IRT inspection is presented showing the detection of hot spots, which reveal the inefficient working conditions of certain regions of the panel.

The application of IRT as an NDT tool has been extended to many industries for the inspection of other elements, such as pipelines [122], solar cells [121] or even concrete [123]. However, it is in the aeronautical sector that IRT for NDT has experienced the greatest increase. The main reason for this situation is the reduced thickness of the components manufactured in this industry, for which weight is limited. As a result, the defects are superficial enough to enable IRT to detect them easily. Many researchers have developed methodologies for detecting cracks in metallic parts of airplanes [124–126], considering different stimulation techniques and also different processing algorithms [127].

The principal defect originated in metallic materials is the crack. This defect can be caused by fatigue load or thermal variations. Studies have demonstrated the efficiency of IRT in the accurate detection of this type of defect. In [128], the detection of surface cracks is analyzed employing laser and optical pulsed IRT with the smallest crack detected of about 5–10 μm. The laser heating technique was concluded to be preferable, as it provides better control, although the laser is a large, heavy and expensive device. On the contrary, flash is cheap and smaller, but reflection issues must be considered. In both cases, IRT was proven to be a suitable technique for automatic inspections, being a competitive alternative to eddy current, penetrant and magnetic particle testing.

**Figure 16.**
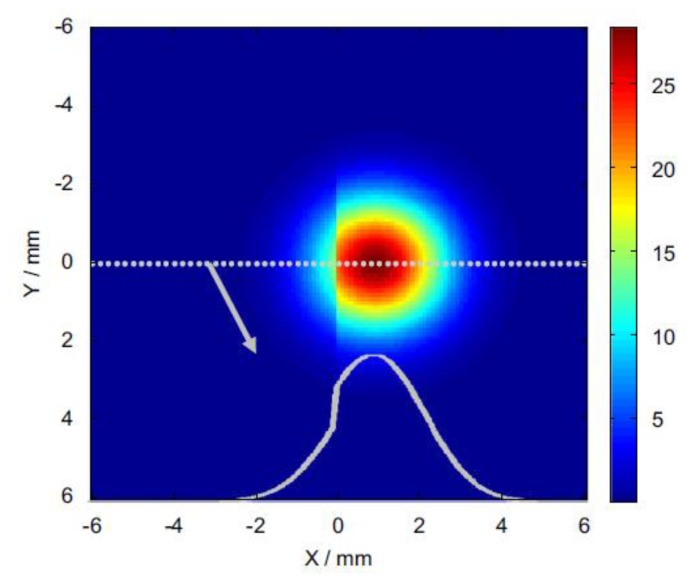
Simulated thermal image of a stainless steel sample containing a 5 mm-long, 1 μm-wide, opening crack (frame with permission from [129]).

Another study conducted with laser spot IRT is [129] in which a 3D simulation is carried out to simulate the heat flow from a laser-heated spot in the proximity of a crack. In Figure 16, the simulated thermal image corresponding to the detection of an open crack by laser thermography and the variation induced in the temperature of the region under influence are observed. A novel second derivative image processing method for extracting images of cracks after raster scanning was also developed. This proved to be highly competitive with regards to most established NDT techniques, such as dye penetrant, with the added value of being non-contact and requiring no surface preparation.

Laser spot is not the only IRT technique for proper crack detection. UET has also been evaluated for this task [130]. This evaluation concludes that UET can effectively detect closed cracks considered undetectable by traditional NDT methods, including optically-stimulated IRT. Thanks to its large area imaging capability, high test productivity and safety, ultrasound IRT is a powerful NDT tool for the inspection of cracks in large aluminum structures. Its disadvantage is the requirement of a coupling element.

**Figure 17.**
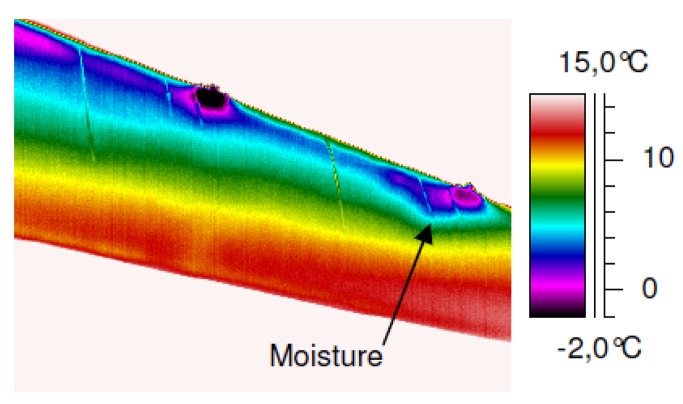
Moisture detected under the upper hinge of the rudder test sample (frame from [132]).

The application of IRT as an NDT tool in the aeronautical sector is not limited to metallic materials [131]. The increasing use of advanced materials, such as composite, for which conventional NDT techniques can be limited or even useless, is a mere example of an application field. These materials are also included within the scope of IRT. In fact, the main capability of detection provided by IRT occurs in composite sandwich structures for water inclusion defects [132]. In this study, the whole structure is cooled below the freezing point of water, then warmed up to room temperature. Phase transition energy, which is needed to melt the penetrated water, can be detected by thermography, owing to the change in temperature that it produces. This defect is quite common in this type of material, where water can penetrate into the core. Once there, the water undergoes cyclic phase changes from liquid to solid, both when the aircraft is flying and when on the ground, causing important breakage in the material, due to expansion effects. Figure 17 shows the efficient detection by IRT of water in a real rudder of an airplane corresponding to the location of colder areas of the surface.

The main defects that are detected by IRT in composite materials are delaminations [133,134], debonding [135,136] and water in sandwich panels. Many investigations were conducted to define methodologies employing different excitation techniques [137,138] and processing algorithms [139].

**Figure 18.**
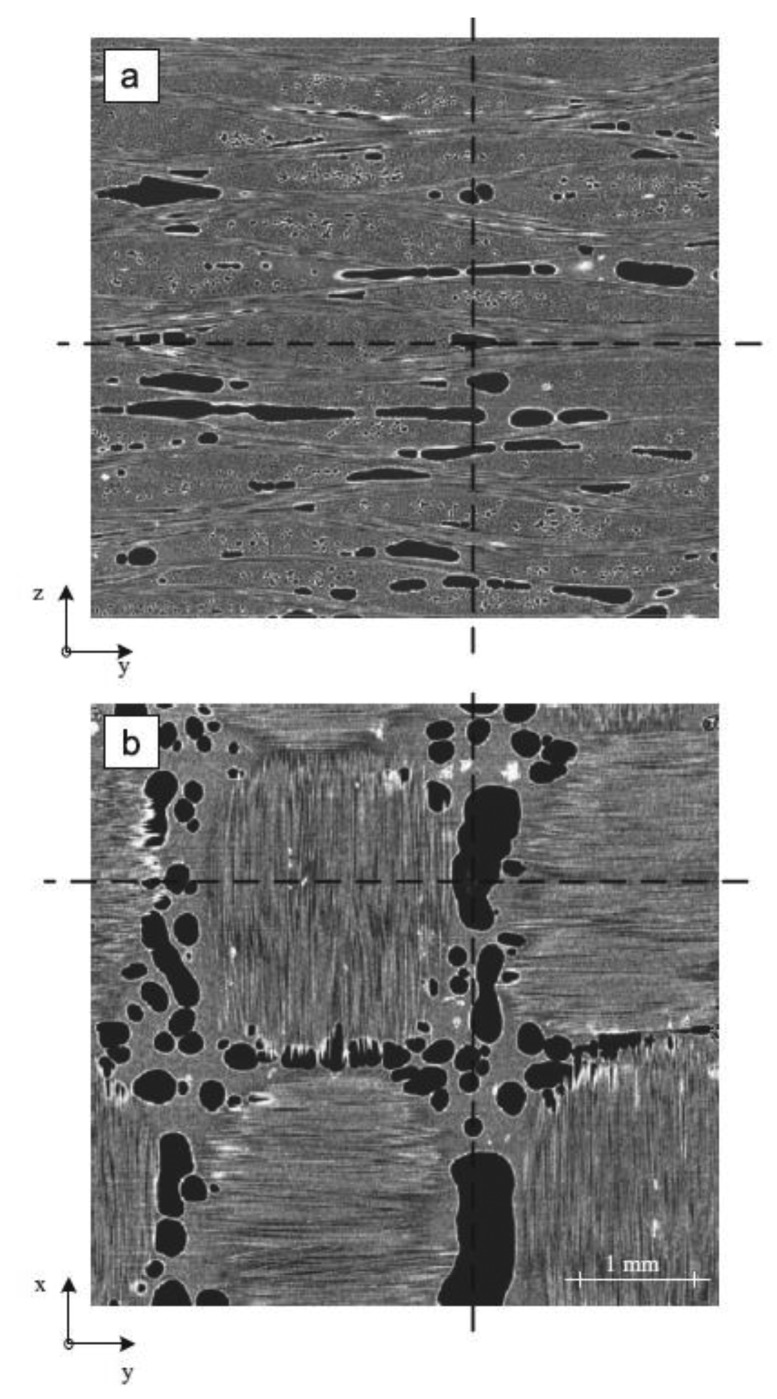
Computed tomography cross-section plot of a typical void structure in a woven CFRP (frame with permission from [140]).

In composite materials, two common defects resulting from incorrect manufacturing processes are excessively high porosity and fiber misalignment. In [140], optical pulsed IRT is evaluated and compared to ultrasonic testing X-ray computed tomography in porosity detection. Figure 18 is the result of an inspection conducted by means of computed tomography, showing the high resolution level provided by this NDT technique and the appearance of the porosity in composite materials. It was concluded that for porosity contents higher than 0.5%, IRT and UT correlate well and that porosity, as well as different shape factors, influence the ultrasonic and thermographic measurement in a similar way. Moreover, some advantages were detected for IRT inspections: it is a non-contact testing method that allows large areas to be tested and gives data that can be interpreted in a reasonably straightforward procedure. In addition, active IRT allows for a rapid non-intrusive inspection of complex-shaped structures and is very fast, thereby very cost efficient.

Closely related to manufacturing defects, like porosity and fiber misalignment, is the important role they play in the generation of more severe defects, such as delaminations. Composite materials are less prone to corrosion and cracking than other materials, such as metals. However, they are extremely sensitive to impact damage. Accidental impacts can cause damage that severely reduces their structural stiffness, creating invisible cracks and delaminations due to the propagation of mechanical energy inside the material. Even imperceptible impacts on the surface can hide important subsurface damage that can lead to severe consequences.

Numerous research works have been carried out to evaluate the capability of IRT for detecting delaminations. In [141], OPT is applied in transmission, as well as in reflection mode to determine diffusivities for the sound and for the damaged areas, giving good results for damage characterization. Important differences of detection capabilities between the two testing configurations were found. In reflection configuration, artificial delaminations can only be detected to a maximum depth of 1.7 mm, whereas in transmission configuration, most artificial delaminations can be detected correctly up to a thickness of 5.9 mm.

However, OPT is not the only technique for the detection of delaminations in composite materials. In [142], eddy current IRT was employed and resulted in comparable results to those obtained with UT, acoustic emission, Bragg gratin sensors (FBG), conventional eddy current, microwave or speckle shearing interferometry. Eddy current IRT combines the advantages of conventional eddy current and thermal wave testing and can be used in both reflection and transmission configurations, providing a higher inspection speed and depth with higher resolution. Unlike OPT, this technique does not depend on the surface conditions, nor does it require a couplant, like UT.

**Figure 19.**
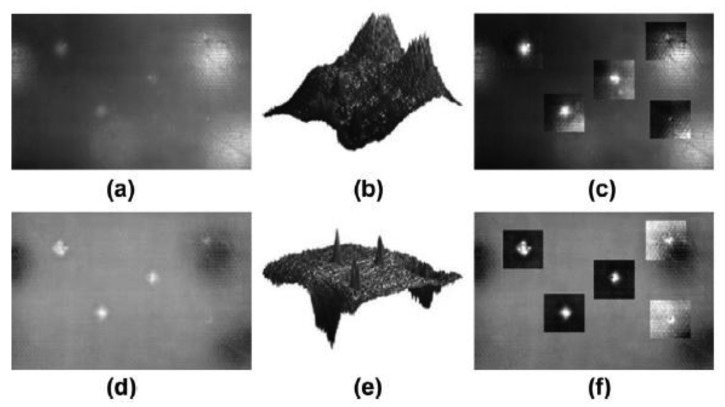
Infrared image enhancement. (**a**) Raw infrared image at t = 20 s; (**b**) 3D representation of the raw infrared image; (**c**) Raw infrared image with enhanced thermal contrast for each defect; (**d**) Phase of the DFT for the minimum frequency; (**e**) 3D representation of the phase; (**f**) Phase of the DFT with enhanced thermal contrast for each defect (frame with permission from [143]).

Additional advances in the detection of delaminations in composite materials can be found in [143], where a method to automatically detect impact damage in CFRP using active thermography and a neural network-based system is proposed. Pulsed phase thermography adapted to optical step heating is applied as an IRT inspection technique, and then, the acquired data is processed. Afterwards, the ROIs are detected using edge detection and clustering, convex hulls and active contours. Several specimens with different features and artificial damage caused by energies from 6 J to 50 J were tested. All defects were correctly detected, demonstrating the proposed procedure to be reliable and robust under different conditions. Figure 19 shows some of the steps used to detect the defects.

**Figure 20.**
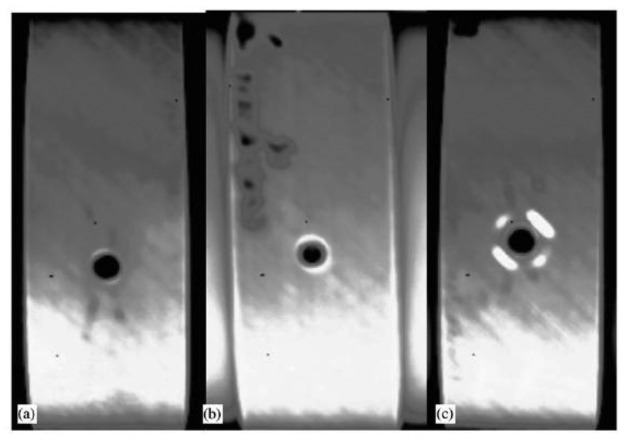
Thermal images of defects in drilled holes (frame with permission from [144]).

In addition to the aforementioned studies focused on specific defects, evaluations of IRT technology for global feasibility assessment in the aircraft industry have also been conducted. An example is [144], where several different real defects, such as notches, drilled holes, impact damage and through skin assessment, were studied by means of pulsed and pulsed phase IRT, taking into account thermal modeling for the evaluation of integrity. Excellent results were obtained in all cases, but the method is highly dependent on the defect depth and size, restricting its application to near-surface defect imaging. In Figure 20, the detection of defects in drilled holes by means of pulsed phase IRT is shown.

## 5. Conclusions

Infrared thermography is a fast, clean and safe technology that is used in a wide variety of applications. This paper has reviewed the use of infrared thermography in two very important fields: temperature measurement and non-destructive testing. The principles and essential theoretical background in these two fields have been reviewed. This background information is provided to help the dissemination of these technologies and to assist beginners in a better understanding of the subject. Moreover, recent work on these topics has been reviewed and discussed.

Infrared thermography has experienced a great evolution in a relatively short time. Important improvements were achieved in different fields. However, there is a variety of limitations that need to be taken into account. Infrared thermography is highly dependent on the sensor selection and the experimental setup. It may be affected by the instrument and by the environment. These problems can be minimized, but only with adequate setup and testing procedures, which mostly depend on the operator's skill.

Infrared thermography is a mature technique for non-destructive testing. Recent advances in this field allow this technology to detect many types of defects. However, a defect can only be detected using infrared thermography if it opposes enough thermal resistance to create visible thermal contrast. Future sensors with improved sensibility are required to improve the general applicability of this technique. Further work is also required in signal and image processing on the acquired infrared thermal images in order to enhance the detection, to simplify the interpretation of the results and to reduce human interference.

Nowadays, infrared sensors are present in many different fields, but their use is still not widespread. This is partially due to the cost and also due to lack of adequate training. However, fast and affordable hardware developed recently indicates that many other fields will take advantage of the use of infrared thermography in the near future, integrating inexpensive infrared sensors in our daily lives.

## Figures and Tables

**Table 1.** SNR values for each defect on the left side of the specimen.

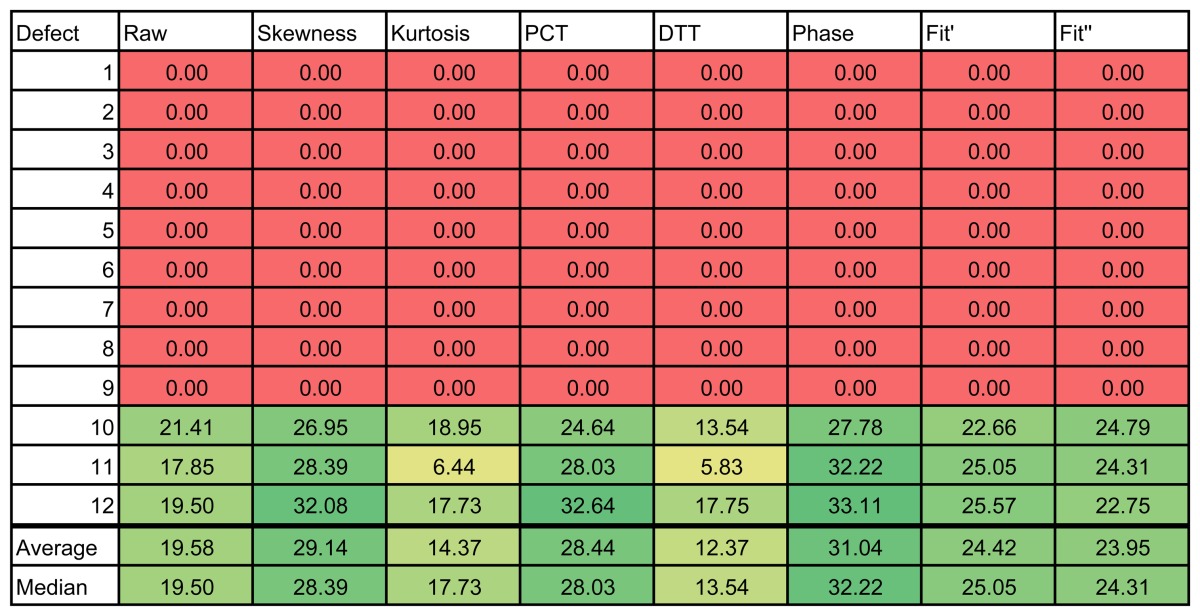

**Table 2.** SNR values for each defect on the right side of the specimen.